# Electrocardiographic characteristics for the prediction of under‐sensing in implantable loop recorders

**DOI:** 10.1002/joa3.12782

**Published:** 2022-09-28

**Authors:** Hirota Kida, Masato Kawasaki, Yoshitaka Kikuchi, Kana Okada, Tetsuya Watanabe, Takahisa Yamada

**Affiliations:** ^1^ Department of Clinical Engineering Osaka General Medical Center Osaka Japan; ^2^ Division of Cardiology Osaka General Medical Center Osaka Japan

**Keywords:** amplitude in anterolateral chest leads, implantable loop recorder, under‐sensing

## Abstract

**Background:**

Under‐sensing (US) in implantable loop recorders (ILRs) interferes with the accurate diagnosis of arrhythmia, but there is little information available on the details of US of ILRs. The aim of this study was to clarify the frequency of US in patients with ILRs and to investigate the predictors of US in ILRs prior to implantation.

**Methods and Results:**

We studied 46 consecutive patients implanted ILR. During the mean follow‐up period of 499 ± 363 days, 15 events of US were observed in five patients. There were no significant differences in patient characteristics between patients with and without US. In *standard 12‐lead electrocardiogram* (ECG), QRS complex amplitude in anterolateral chest leads (V2 to V5) were significantly lower in patients with than without US (V2: 0.88 [0.66, 1.22] mV vs. 1.67 [1.23, 2.29] mV, *p* = .010 V3: 1.25 [1.00, 1.26] mV vs. 1.90 [1.41, 2.29] mV, *p* = .013; V4: 1.14 [0.96, 1.38] mV vs. 1.93 [1.65, 2.64] mV, *p* = .023; V5: 0.57 [0.50, 0.75] mV vs. 1.60 [1.20, 1.98] mV, *p* = .011, respectively). ROC curve analysis showed that cut‐off values of 1.30 mV of QRS complex amplitude in V2, 1.26 mV of that in V3, and 0.75 mV of that in V5 had moderate accuracy for predicting US (V2: sensitivity 68%, specificity 100%, area under the curve [AUC] 0.86; V3: sensitivity 85%, specificity 80%, AUC 0.85; V5: sensitivity 98%, specificity 80%, AUC 0.85, respectively).

**Conclusions:**

US was observed in 10.9% patients with an ILR. QRS complex amplitude in anterolateral chest leads (V2 to V5) on ECG might be useful for predicting US in patients with ILRs.

## Graphical abstract

Under‐sensing (US) of second‐generation implantable loop recorder (ILR) was observed in 10.9% of patients. QRS complex amplitude in anterolateral chest leads on standard 12 lead ECG might be useful for predicting US in patients with second‐generation ILRs.

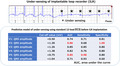

## 1 INTRODUCTION

**FIGURE 1:**
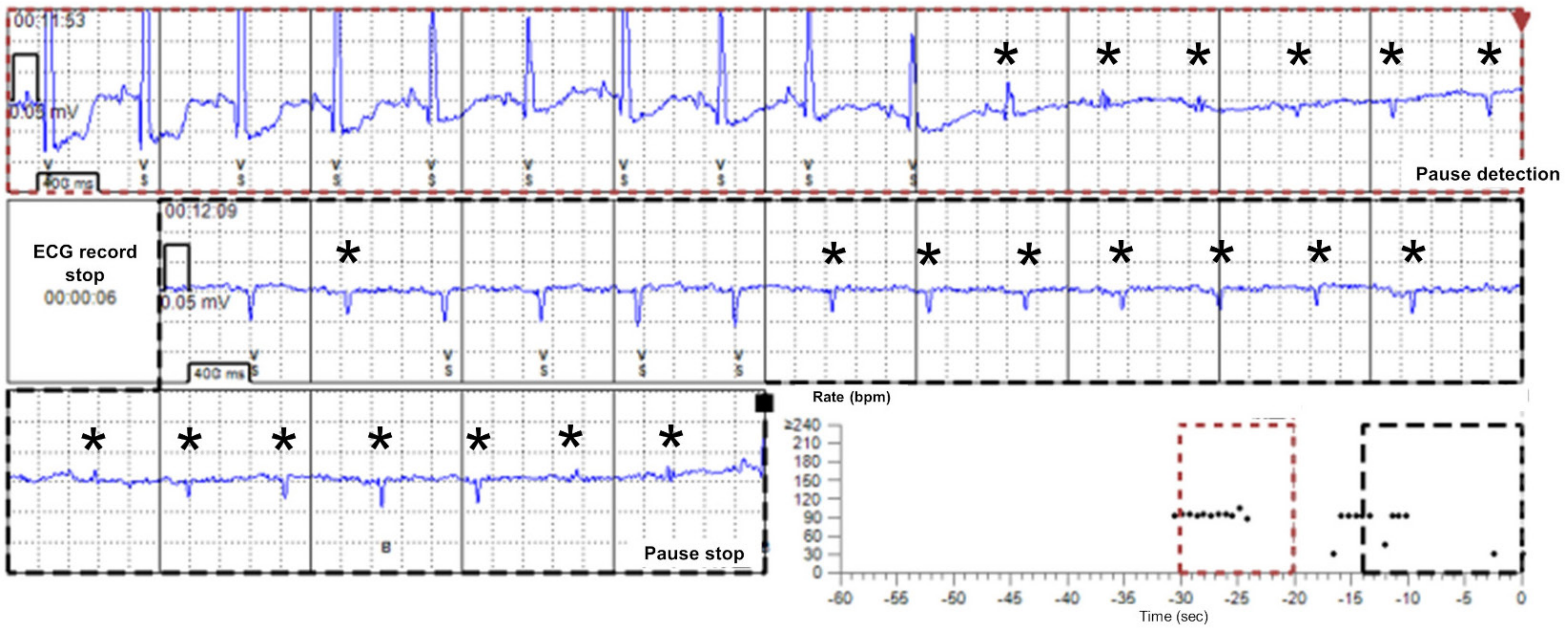
False cardiac arrest events detected by implantable loop recorder. The waves (asterisks) with abrupt decrease of amplitude that occur following a tiny P wave are thought to be under‐sensed QRS waves by the implantable loop recorder.

Implantable loop recorder (ILR) devices are used to continuously monitor the heart's electric activity and employ a subcutaneous bipolar electrogram (EGM) to diagnose heart rhythm disorders.^1^ They are a well‐established tool for evaluating recurrent unexplained syncope^2^, ^3^, ^4^ and cryptogenic stroke.^5^, ^6^, ^7^ It is essential to achieve good signal in order to make a correct diagnosis.^8^ Some extent of under‐sensing (US; Figure 1) has been reported in ILRs,^9^ which interferes with the accurate diagnosis of arrhythmia^10^; however, there is little detailed information available on US of ILRs, especially with respect to second‐generation ILRs. The aim of this study was to clarify the frequency of US in patients with a second‐generation ILR and to investigate the predictors of US of ILR prior to implantation.

## 2 METHODS

### 2.1 Device implantation and programing

Consecutive patients who were implanted with an ILR (Reveal LINQ®; Medtronic plc) between October 2016 and February 2021 were enrolled in this study. Indications for implantation included recurrent syncope of unknown origin and cryptogenic stroke. All ILRs were implanted in the left sternal border of the fourth or fifth intercostal space by a cardiologist, following standard operating procedures. In all cases, R‐wave amplitude of the ILR at implantation was >0.2 mV (as recommended by the manufacturer) and the ILR was programed in the nominal setting: (i) sensitivity, 0.035 mV; (ii) tachycardia detection, >200 beats per minute (BPM) (16 consecutive beats); and (iii) bradycardia detection, <30 BPM (4 consecutive beats), and (iv) pause detection, 3 s.

### 2.2 Data collection

**FIGURE 2:**
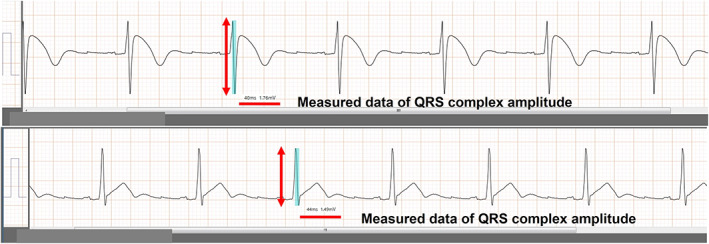
Measurement of QRS complex amplitude in standard 12‐lead *electrocardiogram*.

Patient information was collected from medical records, including age, sex, body mass index (BMI), and medical history. In all patients, echocardiography and standard 12‐lead *electrocardiogram* (ECG) were evaluated before ILR implantation. In echocardiography, left ventricular diastolic diameter, left ventricular ejection fraction, and left atrial diameter were measured by the standard method. Heart angle and ILR angle were manually measured in a chest posteroanterior X‐ray image. The heart angle was defined as the angle between the horizontal line and the line dividing the ventricular area symmetrically via the apex. The ILR angle was similarly defined.^11^ In standard 12‐lead ECG, QRS duration and electrical axis deviation were measured by an electrocardiograph analysis system (MBF‐1000; FUKUDA DENSHI, Inc.). Moreover, QRS complex amplitude in standard 12‐lead ECG was defined as the absolute value of the potential formed from the Q, R, and S waves (Figure 2) and measured by an electrocardiograph analysis system (MBF‐1000; FUKUDA DENSHI, Inc.). Finally, QRS amplitude of the ILR was measured by a pacing programer (Programmer 2090; Medtronic, Inc.) before the patient was discharged.

### 2.3 Follow‐up

All patients were provided with a remote monitoring system (My Care Link; Medtronic) that automatically downloads key data stored by the ILR and sends them to the website daily. Clinical engineering specialists explained the proper operation of the remote monitoring system to the patients before discharge. In the case of arrhythmia events, the clinical engineer reported the event to the electrophysiologist according to the institution's protocol. For evaluation of the proportion of true and false positive arrhythmia detections, all recordings were reviewed by the same electrophysiologist who was responsible for clinical management. The endpoint of this study was US (defined as failure of the device to sense intrinsic R‐wave) of ILRs. No further sensitivity adjustment was made during the follow‐up period. The study protocol was approved by the Ethics Committee of Osaka General Medical Center in accordance with the Helsinki Declaration. Since this study was a retrospective study, we used the opt‐out system according to the ethics guidelines on human medical research by the Japanese government. The device manufacturer did not sponsor or influence the study in any way.

### 2.4 Statistical analysis

Categorical variables are presented as frequencies and compared using the chi‐squared test between groups with or without US. Continuous variables are presented as the mean (standard deviation) or median [interquartile range] and compared using Student's *t*‐test or the Mann–Whitney *U*‐test as appropriate between groups with or without US. Correlations between QRS complex amplitude on standard 12‐lead ECG and the amplitude of the ILR were assessed using Pearson's correlation coefficients. Receiver‐operating characteristic (ROC) curves were drawn for specific parameters and the area under the curve (AUC) was calculated for each. The cut‐off value was defined as the value at which sensitivity and specificity were maximized values by ROC curve analysis for the prediction of US. All analyses were performed using SPSS 27.0 (IBM Corporation) or R software (version 4.0.0; R Foundation for Statistical Computing) with R Studio (version 3.6.1).

## 3 RESULTS

**FIGURE 3:**
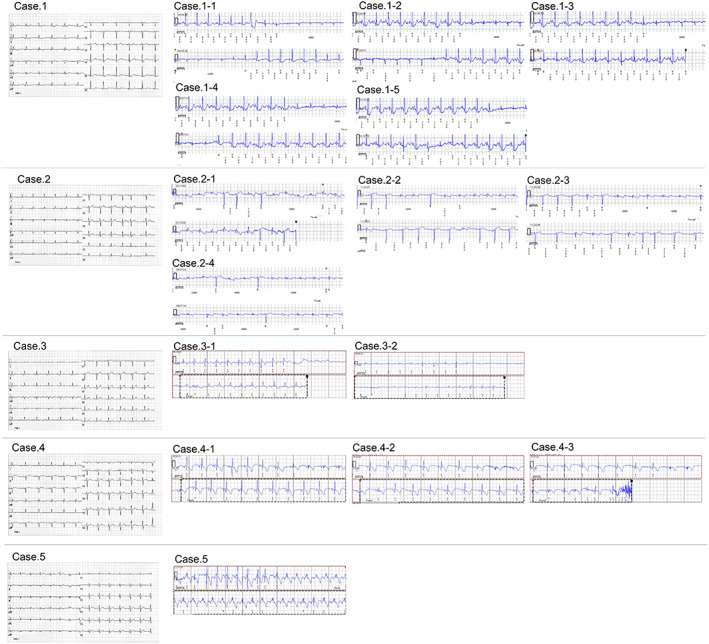
Standard 12‐lead *electrocardiogram and* under‐sensing electrograms in five patients with under‐sensing. Case.1 had five under‐sensing events, Case.2 had four, Case.3 had two, Case.4 had three, and Case.5 had one, respectively. Standard 12‐lead electrocardiogram before ILR implantation and all under‐sensing events in each case were shown. ILR, implantable loop recorder

Forty‐six consecutive patients with ILRs were enrolled in this study. During the mean follow‐up period of 499 ± 363 days, 15 US events were observed in five patients during both the day and night. The time duration from implantation to the first occurrence and the counts of US in five patients were 27 days (five events), 36 days (four events), 44 days (three events), 67 days (two events), and 84 days (one event), respectively. All stored US EGM and standard 12‐lead ECG in five patients with US were shown in Figure 3. The five patients with US did not have dilated cardiomyopathy but have 40% (2/5) old myocardial infarction.

**FIGURE 4:**
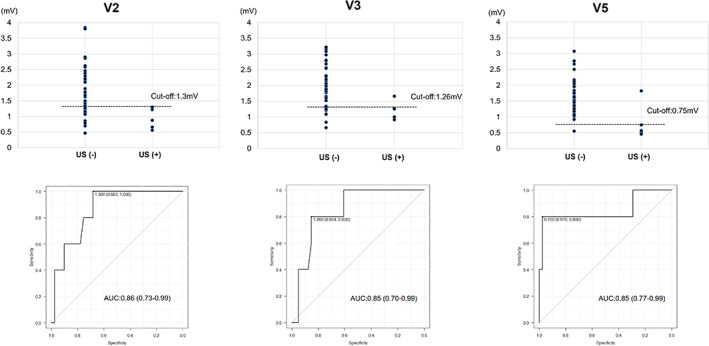
Distribution of QRS complex amplitude and ROC curves for under‐sensing in V2, V3, and V5. ROC, receiver‐operating characteristic; US, under‐sensing

**FIGURE 5:**
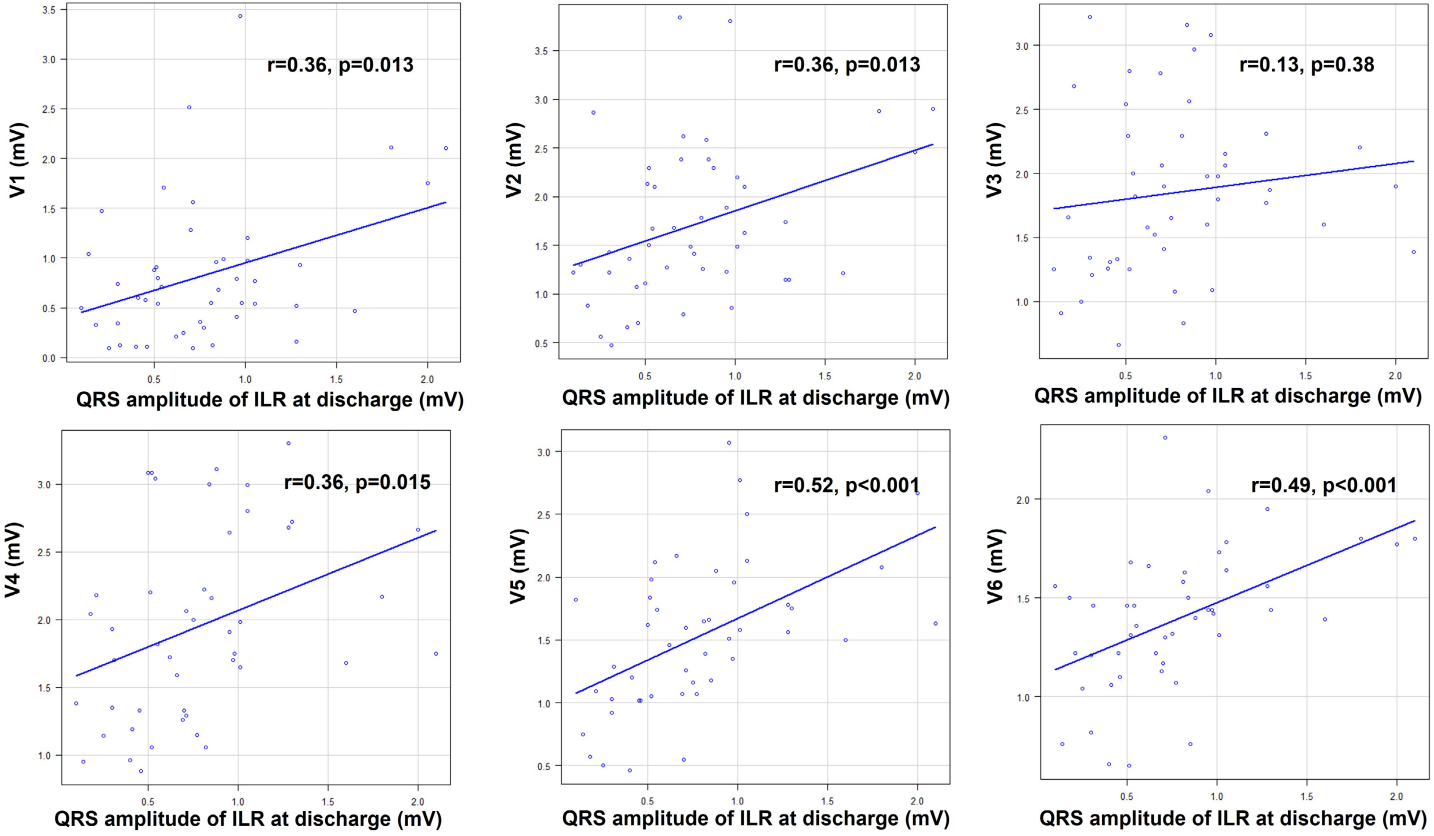
The correlation between QRS complex amplitude in anterolateral chest leads on standard 12‐lead ECG and QRS amplitude of the ILR at discharge. ECG, electrocardiogram; ILR, implantable loop recorder

The baseline clinical characteristics are shown in Table 1. There were no significant differences in age, sex, BMI, underlying heart disease or echocardiographic parameters between patients with and without US. Peak amplitude of the ILR at discharge was significantly lower in patients with US than in those without US. In standard 12‐lead ECG, QRS complex amplitude in anterolateral chest leads (V2 to V5) were significant lower in patients with US than in those without US. Table 2 shows the ROC curve analysis for each model of US for anterolateral chest leads V1 to V6. Each model in leads V2 to V5 was good predictor of US, with AUC greater than >0.8. ROC curve analysis showed that the cut‐off values of 1.30 mV of QRS complex amplitude in V2, 1.26 mV of QRS complex amplitude in V3 and 0.75 mV in V5 had moderate accuracy for predicting US (V2: sensitivity 68%, specificity 100%, AUC 0.86; V3: sensitivity 85%, specificity 80%, AUC 0.85; V5: sensitivity 98%, specificity 80%, AUC 0.85, respectively) (Figure 4). On the other hand, regarding QRS amplitude of ILR at the discharge, ROC curve analysis showed that the cut‐off value: 0.40 mV, sensitivity: 93%, and specificity 100%, AUC: 0.98 (Figure S1). There were mild to moderate correlations between QRS complex amplitude in anterolateral chest leads V1, V2, V4, and V5 on standard 12‐lead ECG and QRS amplitude of the ILR at discharge (V1: *r* = .36, *p* = .013; V2: *r* = .36, *p* = .013; V4: *r* = .36, *p* = .015; V5: *r* = .52, *p* < .001; V6: *r* = .49, *p* < .001) (Figure 5).

**TABLE 1:** Baseline characteristics.

	All patients	Under‐sensing (−)	Under‐sensing (+)	*p* value
Number	46	41	5	
Male (%)	30 (71.4)	27 (73.0)	3 (60.0)	.613
Age (years)	70.0 (12.3)	69.5 (12.4)	74.0 (12.6)	.449
Height (cm)	163.3 [158.2, 169.8]	164.0 [144.0, 179.1]	161.7 [156.0, 173.5]	.756
Weight (kg)	62.1 [54.4, 68.4]	62.6 [37.2, 87.2]	57.0 [53.0, 79.3]	.985
BMI	23.1 [21.3, 24.4]	23.0 [15.6, 31.5]	23.1 [20.5, 26.3]	.712
Underlying heart disease				
OMI, *n* (%)	5 (10.9)	3 (7.3)	2 (40.0)	.084
Dilated cardiomyopathy, *n* (%)	2 (4.3)	2 (4.3)	0 (0)	>.999
Chest PA X‐ray				
Heart angle (degree)	47.0 [43.0, 49.0]	47.0 [43.0, 49.0]	40.5 [37.8, 43.3]	.198
ILR angle (degree)	38.0 [28.0, 57.0]	38.0 [28.5, 56.5]	47.0 [31.0, 63.0]	.970
Echocardiography				
LVDd (mm)	45.5 [43.8, 48.0]	46.0 [38.0, 61.0]	44.0 [40.0, 46.0]	.134
LVEF (%)	68.0 [61.0, 71.8]	67.0 [37.0, 80.0]	68.0 [63.0, 79.0]	.368
LAD (mm)	38.2 [34.3, 41.8]	37.0 [29.0, 44.0]	38.4 [19.0, 56.0]	.574
Standard 12‐lead ECG				
Atrial fibrillation, *n* (%)	3 (6.5)	3 (6.5)	0	>.999
QRS duration (ms)	102 [95, 108]	102 [96, 108]	96 [95, 102]	.437
Electrical axis deviation (°)	38 [9, 60]	38 [10, 55]	15 [−19, 78]	.771
QRS complex amplitude (mV)				
I	0.68 [0.47, 0.90]	0.70 [0.49, 0.91]	0.55 [0.43, 0.56]	.107
II	0.82 [0.57, 0.96]	0.83 [0.59, 1.05]	0.59 [0.32, 0.84]	.150
III	0.57 [0.48, 0.71]	0.56 [0.49, 0.68]	0.73 [0.37, 0.76]	.655
aVR	0.72 [0.50, 0.87]	0.75 [0.57, 0.87]	0.50 [0.49, 0.51]	.039
aVL	0.52 [0.39, 0.72]	0.51 [0.39, 0.72]	0.63 [0.40, 0.72]	.969
aVF	0.55 [0.42, 0.79]	0.54 [0.46, 0.77]	0.68 [0.20, 0.79]	.741
V1	0.64 [0.37, 0.99]	0.71 [0.47, 0.99]	0.33 [0.11, 0.50]	.071
V2	1.50 [1.21, 2.18]	1.67 [1.23, 2.29]	0.88 [0.66, 1.22]	.010
V3	1.81 [1.33, 2.19]	1.90 [1.41, 2.29]	1.25 [1.00, 1.26]	.013
V4	1.87 [1.36, 2.21]	1.93 [1.65, 2.64]	1.14 [0.96, 1.38]	.023
V5	1.60 [1.11, 1.93]	1.60 [1.20, 1.98]	0.57 [0.50, 0.75]	.011
V6	1.43 [1.22, 1.64]	1.44 [1.22, 1.66]	1.04 [0.76, 1.50]	.130
QRS amplitude of ILR at discharge (mV)	0.58 [0.40, 0.77]	0.64 [0.27, 0.89]	0.18 [0.12, 0.26]	<.001

*Note*: Data are presented as values and percentages, mean values ± standard deviation (SD), or medians with first to third quartiles [Q1, Q3].

Abbreviations: BMI, body mass index; ECG, electrocardiogram; ILR, implantable loop recorder; LAD, left atrial diameter; LVDd, left ventricular diastolic diameter; LVEF, left ventricular ejection fraction; OMI, old myocardial infarction; PA, posteroanterior.

**TABLE 2:** The model for under‐sensing in ILR by ROC curve analysis in each anterolateral chest lead

	Cut‐off value (mV)	AUC	95% CI	Sensitivity	Specificity
V1: QRS complex amplitude	0.50	0.74	0.47–1.00	0.71	0.81
V2: QRS complex amplitude	1.30	0.86	0.73–0.99	0.68	1.00
V3: QRS complex amplitude	1.26	0.85	0.70–0.99	0.85	0.80
V4: QRS complex amplitude	1.38	0.82	0.60–1.00	0.76	0.80
V5: QRS complex amplitude	0.75	0.85	0.77–0.99	0.98	0.80
V6: QRS complex amplitude	1.04	0.71	0.41–1.00	0.93	0.60

Abbreviations: ILR, implantable loop recorder; ROC, receiver operating characteristic; AUC, area under the curve: CI, confidence interval.

## 4 DISCUSSION

To the best of our knowledge, the present study is the first to clarify in detail the predictors of US in patients with an ILR. Our results showed that US was observed in 10.9% patients with an ILR, and QRS complex amplitude in anterolateral chest leads on standard 12 lead ECG, which suggests that the QRS amplitude of ILRs at discharge might be useful for predicting US in patients with second‐generation ILRs.

Chrysostomakis et al. reported US episodes in 15 of 32 (47%) patients with a first‐generation ILR.^9^ In contrast, in the present patients with a second‐generation ILR, US was observed in 5 of 46 (10.9%) patients, and we clarified that QRS complex amplitude in anterolateral chest leads (V2, V3, and V5) on ECG might be a useful predictor of US in patients with ILRs.

In first‐generation ILRs, a mapping technique was required to determine the optimal implantation site and signal strength at ILR implantation.^8^ On the other hand, Grubb et al. reported that even a simple anatomical approach without mapping provided good signal strength.^12^ Hence, ILR manufacturers now recommend ILR implantation in the left sternal border of the fourth intercostal space without pre‐mapping. In the present study, however, US was observed in five patients (10.9%) by remote monitoring, despite amplitude of 0.2 mV or more at implantation in all patients as recommended (left sternal border of the fourth or fifth intercostal space), during a mean follow‐up period of 499 ± 363 days. It is possible that amplitude may have been sufficient at the time of implantation and that US might have occurred in the chronic phase. Because US decreases the diagnostic accuracy of ILRs, careful observation by remote monitoring is required. The present results might be useful for identifying patients at high risk of US before implantation.

Under‐sensing interferes with the accurate diagnosis of arrhythmia^10^ and causes an increase in inappropriate remote monitoring alerts, which increases the difficulty of providing individual responses.^13^ Ahn et al. reported that the ILR insertion angle, aVR QRS amplitude, Holter lead V5 QRS amplitude, age, and *left ventricular hypertrophy* (LVH) were associated with the QRS amplitude of ILR.^11^ On the other hand, in our study, amplitude in anterolateral chest leads (V2, V3, and V5) on ECG were associated with US of ILRs, which included the similar results reported by Ahn et al. van Dam et al. also reported that subcutaneous ECG at the implantation site was highly similar to bipolar ECGs on the most proximal part of the body surface.^14^ Therefore, in patients who have lower QRS complex amplitude in anterolateral chest leads V2 to V5 (especially V5) on standard 12‐lead ECG, the amplitude of the ILR might also be lower. It might be necessary to consider multiple implantation sites by mapping sufficient strength voltage before ILR implantation to avoid US. However, in our study the other factors such as ILR insertion angle, aVR QRS amplitude, age, and LVH reported by Ahn et al showed no association with US. These results might be due to the small number of US group. Therefore, large‐scale research of ILR might be required to clarify the details of the cause of US.

In our study, the peak amplitudes of ILRs at discharge were significantly lower in patients with than without US. Previous studies have reported that US could not be reproduced by changing body position or by manipulation of the device in the pocket.^9^, ^10^ Thus, we had hypothesized that patients with low amplitude of the ILR might experience US due to unusual body positions or certain activities. However, US events were observed both day and night in our study, which offers no clues to identifying the cause.

Our study has several limitations. First, our study was conducted at a single‐center and a included a small number of subjects, so it might seem difficult to draw a conclusion in this small sample size of only five patients with the clinical event. Second, QRS amplitude of ILR at the implantation is important, but their accurate values were not obtained, although we confirmed that QRS amplitudes of ILR at the implantation were 0.2 mV or more. Therefore, it was not possible to investigate the relationship between these data and US. Third, we analyzed only Reveal LINQ®, and the results for other ILR models are unknown. Therefore, the results of our study cannot be generalized to all patients in whom an ILR has been implanted. So, a multi‐center observational study that includes a larger number of subjects is required in the future.

## 5 CONCLUSION

Under‐sensing was observed in 10.9% (5/46) of patients with ILRs. QRS complex amplitude in anterolateral chest leads V2 to V5 on standard 12‐lead ECG and the peak amplitude of the ILR at discharge might be useful for predicting US in patients with ILRs.

## AUTHOR CONTRIBUTIONS

Masato Kawasaki, Tetsuya Watanabe and Takahisa Yamada participated in the design of the study and coordination and helped to draft the manuscript. Yoshitaka Kikuchi, Kana Okada carried out data collection. All authors read and approved the final manuscript.

## CONFLICT OF INTEREST

All authors declare no conflicts of interest associated with this manuscript.

## ETHICS APPROVAL STATEMENT

This study was approved by the Ethics Committee of Osaka General Medical Center in accordance with the Helsinki Declaration.

## PATIENT CONSENT STATEMENT

Since this study was a retrospective study, we used the opt‐out system according to the ethics guidelines on human medical research by the Japanese government.

## CLINICAL TRIAL REGISTRATION

Because of the retrospective study, this study was not registered for clinical trial registration.

## Supporting information


Figure S1

## References

[1] Task Force Members , Brignole M , Vardas P , Hoffman E , Huikuri H , Moya A , et al. Indications for the use of diagnostic implantable and external ECG loop recorders. Europace. 2009;11:671–87. 19401342 10.1093/europace/eup097

[2] Farwell DJ , Freemantle N , Sulke AN . Use of implantable loop recorders in the diagnosis and management of syncope. Eur Heart J. 2004;25:1257–63. 15246645 10.1016/j.ehj.2004.03.010

[3] Sivakumaran S , Krahn AD , Klein GJ , Finan J , Yee R , Renner S , et al. A prospective randomized comparison of loop recorders versus Holter monitors in patients with syncope or presyncope. Am J Med. 2003;115:1–5. 12867227 10.1016/s0002-9343(03)00233-x

[4] Ng E , Stafford PJ , Ng GA . Arrhythmia detection by patient and auto‐activation in implantable loop recorders. J Intervent Cardiac Elctrophysiol. 2004;10:147–52. 10.1023/B:JICE.0000019268.95018.91 15014215

[5] Gladstone DJ , Spring M , Dorian P , Panzov V , Thorpe KE , Hall J , et al. Atrial fibrillation in patients with cryptogenic stroke. N Engl J Med. 2014;370:2467–77. 24963566 10.1056/NEJMoa1311376

[6] Svennberg E , Engdahl J , Al‐Khalili F , Friberg L , Frykman V , Rosenqvist M . Mass screening for untreated atrial fibrillation: the STROKESTOP study. Circulation. 2015;131:2176–84. 25910800 10.1161/CIRCULATIONAHA.114.014343

[7] Hart RG , Diener HC , Coutts SB , Easton JD , Granger CB , O'Donnell MJ , et al. Cryptogenic stroke/ESUS international working group. Embolic strokes of undetermined source: the case for a new clinical construct. Lancet Neurol. 2014;13:429–38. 24646875 10.1016/S1474-4422(13)70310-7

[8] Zellerhoff C , Himmrich E , Nebeling D , Przibille O , Nowak B , Liebrich A . How can we identify the best implantation site for an ECG event recorder? Pacing Clin Electrophysiol. 2000;23:1545–9. 11060877 10.1046/j.1460-9592.2000.01545.x

[9] Chrysostomakis SI , Klapsinos NC , Simantirakis EN , Marketou ME , Kambouraki DC , Vardas PE . Sensing issues related to the clinical use of implantable loop recorders. Europace. 2003;5:143–8. 12633638 10.1053/eupc.2002.0301

[10] Chrysostomakis SI , Simantirakis EN , Marketou ME , Vardas PE . Implantable loop recorder undersensing mimicking complete heart block. Europace. 2002;4:211–3. 12135256 10.1053/eupc.2002.0229

[11] Ahn JH , Ryu H , Oh IY , Cho Y , Lee JH . Analysis of the determining factors of detectable P‐wave and amplitude of QRS complex sensed by implantable loop recorder. J Arrhythm. 2021;37:1069–76. 34386134 10.1002/joa3.12582 PMC8339105

[12] Grubb BP , Welch M , Kanjwal K , Karabin B , Kanjwal Y . An anatomic‐based approach for the placement of implantable loop recorders. Pacing Clin Electrophysiol. 2010;33:1149–52. 20353412 10.1111/j.1540-8159.2010.02747.x

[13] Maines M , Zorzi A , Tomasi G , Angheben C , Catanzariti D , Piffer L , et al. Clinical impact, safety, and accuracy of the remotely monitored implantable loop recorder Medtronic reveal LINQTM. Europace. 2018;20:1050–7. 29016753 10.1093/europace/eux187

[14] van Dam PM , van Oosterom A . Analysing the potential of Reveal for monitoring cardiac potentials. Europace. 2007;9(Suppl 6):vi119–23. 17959687 10.1093/europace/eum216

