# Supplementary figures and images for: Electrocardiographic characteristics for the prediction of under‐sensing in implantable loop recorders
Source: J Arrhythm. 2022 Sep 28;38(6):1063–9. doi: 10.1002/joa3.12782 (PMC9745473; doi:10.1002/joa3.12782)

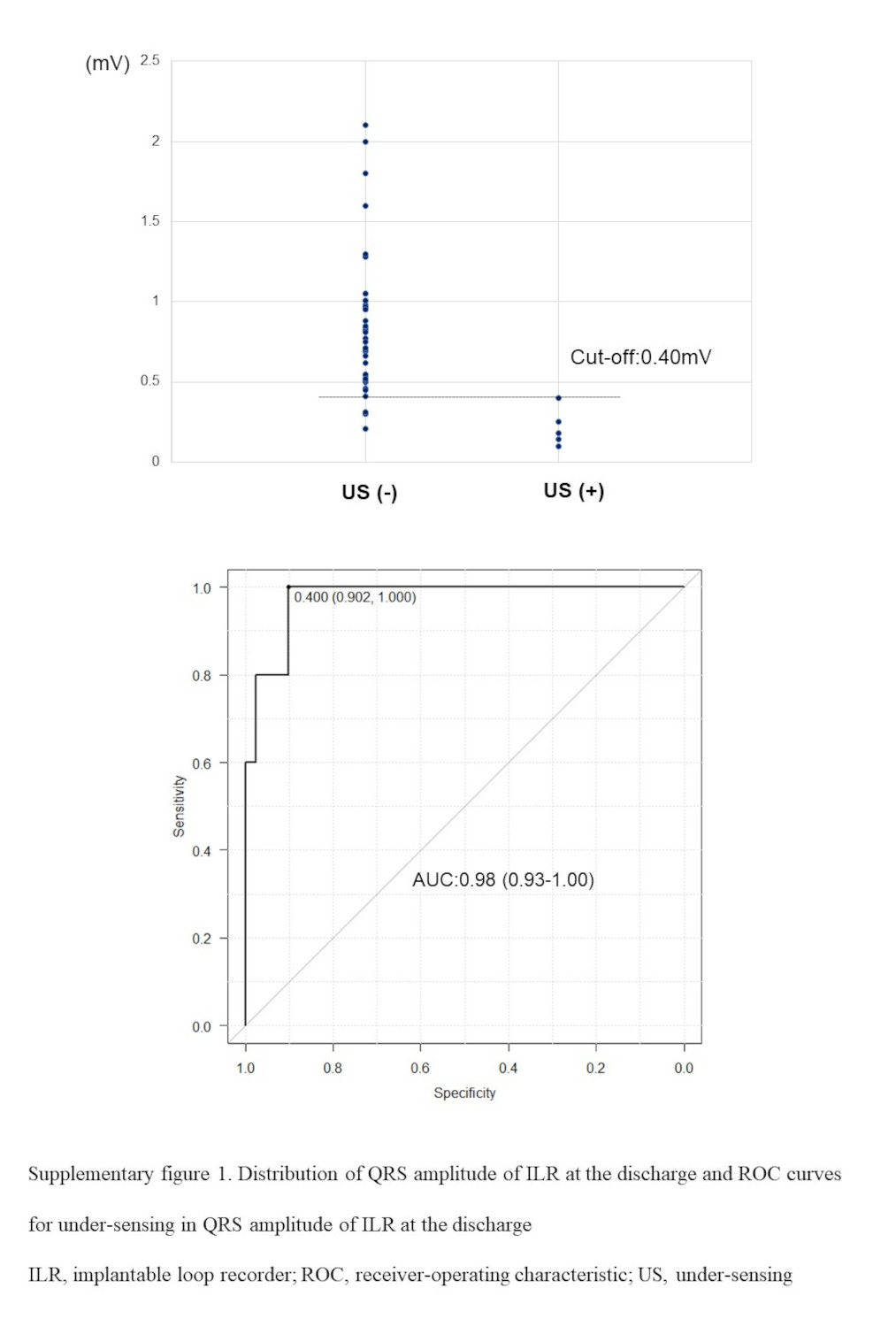

Supplement: Supplementary file 1 — Figure S1 [file JOA3-38-1063-s001.jpg]
